# Donor Cell Acute Myeloid Leukemia after Hematopoietic Stem Cell Transplantation for Chronic Granulomatous Disease: A Case Report and Literature Review

**DOI:** 10.3390/genes14112085

**Published:** 2023-11-16

**Authors:** Giovanni Micheloni, Annalisa Frattini, Marta Donini, Stefano Dusi, Anna Leszl, Annamaria Di Meglio, Martina Pigazzi, Antonio Musio, Marco Zecca, Tommaso Mina, Marco Rabusin, Pamela Roccia, Paolo Bernasconi, Irene Dambruoso, Antonella Minelli, Giuseppe Montalbano, Francesco Acquati, Giovanni Porta, Roberto Valli, Francesco Pasquali

**Affiliations:** 1 Genetica Umana e Medica, Dipartimento di Medicina e Chirurgia, Università dell’Insubria, 21100 Varese, Italy; 2 Istituto di Ricerca Genetica e Biomedica, CNR, 20090 Milano, Italy; 3 Section of General Pathology, Department of Medicine, University of Verona, 37134 Verona, Italy; 4 Clinica Oncoematologica, Dipartimento di Salute della Donna e del Bambino, Università degli Studi di Padova, 35131 Padova, Italy; 5 Istituto di Tecnologie Biomediche, Consiglio Nazionale delle Ricerche, 56124 Pisa, Italy; 6 Pediatric Hematology/Oncology, Fondazione IRCCS Policlinico S. Matteo, 27100 Pavia, Italy; 7 Emato-oncologia e Centro Trapianti, IRCCS Burlo Garofolo, 34137 Trieste, Italy; 8 Department of Molecular Medicine, University of Pavia, 27100 Pavia, Italy; 9 Hematology Department, Fondazione IRCCS Policlinico San Matteo, 27100 Pavia, Italy; 10 Dipartimento di Biotecnologie e Scienze della Vita, Università dell’Insubria, 21100 Varese, Italy; 11 Centro di Medicina Genomica, Università dell’Insubria, 21100 Varese, Italy

**Keywords:** donor cell leukemia, chronic granulomatous disease, acute myeloid leukemia, hematopoietic stem cell transplantation

## Abstract

The patient reported here underwent hematopoietic stem cell transplantation (HSCT) due to chronic granulomatous disease (CGD) caused by biallelic mutations of the NCF1 gene. Two years later, he developed AML, which was unexpected and was recognized via sex-mismatched chromosomes as deriving from the donor cells; the patient was male, and the donor was his sister. Donor cell leukemia (DCL) is very rare, and it had never been reported in patients with CGD after HSCT. In the subsequent ten years, the AML relapsed three times and the patient underwent chemotherapy and three further HSCTs; donors were the same sister from the first HSCT, an unrelated donor, and his mother. The patient died during the third relapse. The DCL was characterized since onset by an acquired translocation between chromosomes 9 and 11, with a molecular rearrangement between the MLL and MLLT3 genes—a quite frequent cause of AML. In all of the relapses, the malignant clone had XX sex chromosomes and this rearrangement, thus indicating that it was always the original clone derived from the transplanted sister’s cells. It exhibited the ability to remain quiescent in the BM during repeated chemotherapy courses, remission periods and HSCT. The leukemic clone then acquired different additional anomalies during the ten years of follow-up, with cytogenetic results characterized both by anomalies frequent in AML and by different, non-recurrent changes. This type of cytogenetic course is uncommon in AML.

## 1. Introduction

Hematopoietic stem cell transplantation (HSCT) is an effective treatment for many hematological diseases, and it is mostly used in malignancies. A major problem is relapse, which may be observed in some patients. In most cases, relapse exhibits the same characteristics as the primary diagnosis, with very few patients developing a different malignant disease in bone marrow (BM) donor cells [1]. Isolated cases in which relapse occurs in donor cells have been reported since 1971, as demonstrated via sex chromosomes in cases of sex-mismatched donors [2] or via the analysis of DNA molecular markers [1]. The development of donor cell leukemia (DCL) is quite rare: Ruiz-Argüelles and co-workers collected a total of 41 reported cases in 2007 [3], Wiseman 64 cases in 2011 [1], Williams and co-workers 162 in 2021 [4]. A review of all the methods used to confirm donor origin was offered by Wiseman [1]. This latter paper also offers a review of all possible etiologic mechanisms of DCL.

We report a patient who underwent HSCT not to treat leukemia or lymphoma, as in the majority of HSCT procedures, but chronic granulomatous disease (CGD, OMIM #306400) and then developed acute myeloid leukemia (AML) in the donor cells. CGD is a primary immunodeficiency that results in severe and life-threatening infections in affected children. It may be caused by mutations in several genes (NCF1, NCF2, NCF4, CYBA, CYBB, CYBC1) encoding the structural or regulatory subunits of the phagocyte NADPH oxidase complex. The most frequent defect is a hemizygous mutation of the CYBB gene, located in the X chromosome (65% of the cases). The other causative mutations lead to autosomal recessive inheritance, and the most frequent are those involving NCF1 (25% of the cases), CYBA (5–10%) and NCF2 (5–10%) [5]. The NCF1 gene, which encodes the subunit p47phox, is located on chromosome 7q11.23; in the same chromosomal region, two pseudogenes (NCF1B and NCF1C) are co-localized, with >99% homology between them and NCF1 [6]. Treatment for CGD patients is generally based on symptomatic approaches or, in more severe cases, on HSCT [7,8,9].

The clinical history of the patient reported here led him to undergo four HSCTs with different donors due to the subsequent relapses of the AML developed in donor cells after the first HSCT; these were characterized by long-lasting clonal chromosome anomalies and clonal evolution.

## 2. Materials and Methods

### 2.1. Clinical Report

The patient was a male, born after an uneventful pregnancy in 2000 in Egypt. The parents were consanguineous (second cousins). He had a younger sister. In the first years of life, he suffered from recurrent bacterial infections (lymphadenitis, osteomyelitis) that led to a diagnosis of chronic granulomatous disease (CGD) when he was four years old. To ascertain the diagnosis, a nitroblue tetrazolium test (NBT) was performed, which was negative, as was the production of superoxide anions by neutrophils after stimulation with phorbol-myristate-acetate (PMA) and N-formyl-L-methionyl-L-leucyl-phenylalanine (fMLP) (see Section 2.3). The consanguinity of the parents suggested an autosomal recessive condition, and in fact they produced only a partial amount of superoxide anions, being heterozygous. The sister, as a normal producer, was shown not to bear the causative mutation. Lung aspergillosis was diagnosed in 2007, and the patient was treated with antimycotic drugs and surgery.

The patient underwent HSCT in September 2008, with the HLA-identical sister as donor, at the Burlo Garofalo Pediatric Hospital in Trieste. The procedure was standard (Supplementary File S1), and donor cells engrafted efficiently. In May 2010, a diagnosis of AML (FAB M4 type) was made. Cytogenetic results showed that AML had developed in the donor cells with XX sex chromosomes and was characterized by a t(9;11) translocation with the fusion of MLL (alias KMT2A) and MLLT3 genes, as well as by an additional chromosome anomaly. Chemotherapy was administered according to the AIEOP LAM 2002 protocol. In October 2010 a complete morphological remission was achieved, but molecular remission was not complete. A second HSCT was performed (data in Supplementary File S1), with the same sister from the first HSCT as the donor. BM monitoring in February 2011 showed 98% of the BM cells having XX sex chromosomes and exhibiting weak positivity for the rearrangement due to the translocation mentioned above. Chronic graft-versus-host (GvHD) disease developed in 2011 (skin and liver).

Growth hormone deficiency was diagnosed in 2012, and replacement therapy was given.

In September 2012, AML relapsed in the BM and central nervous system (CNS). Therapy according to a protocol for relapsed AML was administered, and remission was achieved, although the patient’s clinical condition was complicated by infections due to Escherichia coli and Aspergillus, which were easily treated. After remission, a third HSCT was performed in December 2012 with non-consanguineous donor cells from a placenta; HLA compatibility in this case was 4/6 (data in Supplementary File S1). Some complications arose (lung aspergillosis, gastrointestinal and hepatic GvHD), but remission was maintained in the subsequent years, with complete morphological BM normalization and negative molecular results for the MLL/MLLT3 rearrangement. In March 2018 AML relapsed with localization in the right epididymis, which was surgically removed; testicle biopsies revealed no lesions. The relapse also involved the BM and the CNS, and the molecular rearrangement reappeared. In April 2018, he was admitted to the Pediatric Oncohematology Division of the Policlinico S. Matteo of Pavia, where the situation was re-evaluated, and the relapse was confirmed at the BM and CNS levels. The morphological examination of the BM showed 50–60% blasts. Further chemotherapy cycles were administered, and remission was achieved. In June 2018, the patient underwent a fourth HSCT, with the haploidentical mother as the donor, after depletion of the T TCR α/β+ and B CD19+ lymphocytes as a prophylaxis for GvHD (data in Supplementary File S1). The engraftment was monitored in the following months via chimerism analysis of the BM using PCR in a panel of polymorphisms, and the BM cells were shown to be donor cells from August 2018 to January 2020. In this period, the BM showed complete morphological remission and an absence of the MLL/MLLT3 rearrangement (sensitivity 10-5).

In January 2020 the proportion of donor cells in the BM began to lower, first to 95% and then, in February, to 75%. A further relapse then took place; a molecular analysis of the BM showed the reappearance of the MLL/MLLT3 rearrangement, and chromosome analysis revealed the reappearance of the abnormal clones with further clonal evolution. The patient died due to sepsis in February 2020.

### 2.2. Neutrophils Isolation

Peripheral neutrophil cells were purified via Ficoll–Hypaque separation density-gradient centrifugation (Amersham Pharmacia Biotech, Uppsala, Sweden) from 5 to 10 mL heparinized blood. The granulocytic fraction was subjected to dextran sedimentation followed by erythrocyte hypotonic lysis (NH_4_Cl 0.829%, EDTA 0.125 mM, NaHCO_3_ 0.1%) and washing with PBS.

### 2.3. Superoxide Anion Production

O_2_^−^ release was estimated via cytochrome c reduction: 2 × 10^5^ neutrophils were resuspended in HBSS pH 7.4 containing 80 µM ferricytochrome C type III (Sigma, St. Louis, MO, USA) and stimulated with 100 nM fMLP or 20 ng/mL PMA (Sigma) for 30 min. Cytochrome c reduction was evaluated at 550 nm using the ELx808 Absorbance Microplate Reader (Biotek Instruments Inc.; Winooski, VT, USA).

### 2.4. Electrophoresis and Immunoblotting

Neutrophils were suspended in HBSS (pH 7.4) containing 1 mM di-isopropylfluorophosphate (DFP; Sigma, St. Louis, MO, USA). After 5 min, this solution was discarded, and the cells were lysed with an electrophoresis sample buffer (60 mM Tris/HCl, 20% [*v*/*v*] glycerol, 4% [*w*/*v*] SDS, 2% [*v*/*v*] 2-mercaptoethanol, pH 6.8) and boiled for 5 min. Total cell lysates were subjected to sodium dodecyl sulfate/polyacrylamide gel electrophoresis (SDS/PAGE) on 12% gels and then transferred to nitrocellulose membranes (Amersham). The blots were then rinsed in TBS-T (50 mM Tris, 170 mM NaCl, 0.2% [*v*/*v*] Tween 20; pH 7.5) and incubated for 60 min in TBS-T containing 5% BSA (pH 7.5) (blocking buffer) before overnight incubation (4 °C) with anti-p47phox antibodies (A-7 sc-17844 Santa Cruz Biotechnology, Santa Cruz, CA, USA) diluted 1:500 in TBS-T containing 1 mg/mL BSA. The blots were incubated with horseradish peroxidase-conjugated anti-mouse IgG (Amersham). Bound antibodies were detected via enhanced chemiluminescence western blotting (WB) using detection reagents (Amersham) as previously described [10].

The western blot was performed for the sole purpose of ascertaining whether p47phox was absent in the patient’s blood and to confirm the diagnosis of CGD suggested by the absence of superoxide anion production. For this purely diagnostic purpose, a leukocyte lysate from the patient’s mother was used as a control. Normalization with β actin was not performed as protein quantification was not necessary.

### 2.5. NCF1 Mutation Analysis

The diagnosis of CGD and the loss of the p47 subunit of neutrophil NADPH oxidase (see Section 3), as shown by WB, led us to perform a molecular analysis of the NCF1 gene (a.c. number NM_000265.7).

The genomic DNA of the proband and his mother was extracted from the lysate prepared in 2008, and this was used for WB. The DNA obtained was of good quality but the molecular weight was low.

Molecular analysis of the NCF1 gene is complicated by the presence of 2 pseudogenes that show >99% homology with the gene, as remarked in the introduction. To overcome this problem and to avoid co-amplifying the gene and pseudogenes, Noack and co-workers used an allele-specific long PCR [11]. Unfortunately, the low molecular weight of the DNA obtained did not allow us to perform long PCR as suggested. Instead, we amplified the regions encompassing the 11 exons of the gene using specific primers with the PCR condition as reported by Noack and co-workers [11], taking co-amplification of the gene and two pseudogenes into consideration.

### 2.6. Minimal Residual Disease and HSCT Monitoring

The presence of the MLL/MLLT3 rearrangement was monitored via RT-PCR, according to routine methods.

Chimerism post-HSCT was monitored via multiplex PCR of 16 short tandem repeats (STR) using the PowerPlex 16HS system (Promega, Madison, WI, USA).

### 2.7. Cytogenetics

Chromosome analyses were performed using routine methods on direct preparations of the BM and on 24–48 h cultures by means of the QFQ-banding technique.

Fluorescent in situ hybridization (FISH) was carried out according to standard techniques on BM nuclei and mitoses using the following probes: LSI CEP Y Spectrum Orange/CEP X Spectrum Green Probes (Vysis, Abbott Laboratories, Abbott Park, IL, USA) for investigating XX or XY sex chromosome constitution; LSI MLL dual color (Vysis) for detecting the rearrangement of the MLL gene; and LSI TP 53/CEP 17 (Vysis) for obtaining structural information on the isochromosome of the long arm of chromosome 17 when this anomaly appeared.

Array-based comparative genomic hybridization (a-CGH) was performed on DNA from BM samples using the 244 K genome-wide system (Agilent Technologies Inc., Santa Clara, CA, USA), according to the manufacturer’s instructions.

## 3. Results

The study of NADPH oxidase via the production of superoxide anions after stimulation of isolated neutrophils with fMLP or PMA (Supplementary File S2, Figure S1) and the WB of the p47phox (NCF1) subunit (Supplementary File S2, Figure S2) confirmed that the CGD of our patient was autosomal recessive due to the lack of the p47phox subunit encoded by the NCF1 gene; this is the case for around 25% of the patients [5].

Genetic analysis showed the presence of the c.G579A (p.W193X) mutation in exon 7, carried by the patient and the mother (the DNA of the father was not available, which prevented us from confirming the mutation in the paternal allele). Due to the co-amplification of the NCF1 gene and the two pseudogenes (NCF1B and NCF1C), the exon 7 chromatogram showed a double peak for both the patient and his mother: the base G carried by the WT allele and pseudogenes, and the base A carried by the mutated allele/alleles. Based on the relative heights of the peaks and on the consanguinity of the parents, we have to postulate that this mutation is present in the homozygous state in the patient and is responsible for the GCD.

Table 1 summarizes all the results available from chromosome analyses, FISH and molecular analyses obtained via RT-PCR on BM material after the development of AML.

In May 2010, chromosome analysis and FISH showed that AML had developed in the XX donor cells, which were 93% of the BM cells, and presented with clonal chromosome anomalies, including a translocation between chromosomes 9 and 11 with the involvement of the MLL gene; a consistent clone (21/28 cells) showed balanced translocation between chromosomes 2 and 7 in addition to the t(9;11) translocation (Table 1; Supplementary File S2, Figure S3A). The BM was then monitored using FISH to ascertain the proportion of XX and XY cells and the presence of the MLL/MLLT3 rearrangement due to the translocation. The MLL rearrangement was also monitored via molecular analysis using RT-PCR (Table 1).

In October 2010, despite a complete morphological remission, molecular remission was not complete: nested PCR showed the MLL/MLLT3 rearrangement in one cell out of 102-3. In February 2011, BM monitoring still revealed 98% BM cells with XX sex chromosomes and weak positivity for the rearrangement (1/104-5 positive cells) (Table 1). The BM condition remained unchanged until December 2012: positive for the MLL/MLLT3 rearrangement at the molecular level, but with the rearrangement not detectable via FISH (Table 1). After the third HSCT, both FISH and RT-PCR showed negative results via both FISH and RT-PCR from March 2013 to December 2014 (Table 1).

During the relapse of April 2018, FISH indicated 126/300 nuclei (42%) and 2/17 mitoses with the XY karyotype and 174/300 nuclei and 15/17 mitoses with the XX karyotype: the XX cells bore the t(9;11) translocation already detected, along with additional anomalies not identical to those found in 2010, thus indicating a clonal evolution. The anomalies in addition to the t(9;11) translocation now represented an unbalanced translocation between chromosomes 2 and 10, with a partial monosomy of the long arm of chromosome 2, a partial trisomy of the long arm of chromosome 10 and a dicentric isochromosome of the long arm of chromosome 17 (Table 1, Supplementary File S2, Figure S3B). These results were confirmed and defined precisely via a-CGH, which showed the breakpoint of chromosome 17 resulting in the dicentric isochromosome at band p11.2 at the bp 18,928,347 level (Supplementary File S2, Figure S4).

After remission and a fourth HSCT performed in June 2018 in which the mother was the donor, the BM cells were shown to be of donor origin for August 2018–January 2020, and the molecular rearrangement previously followed-up was not detectable. A relapse took place January–February 2020. The rearrangement reappeared, and the karyotype again showed different anomalies in addition to the t(9;11) translocation. Two clones with these additional anomalies were present, one with a deletion in the short arm of chromosome 12 and with the unbalanced translocation between chromosomes 2 and 10 already detected in 2018; the other clone exhibited the same anomalies along with the dicentric isochromosome of the long arm of chromosome 17 detected in 2018 (Table 1).

## 4. Discussion

The occurrence of DCL has been reported since 1971 [2], but it is very rare. In 2021 a comprehensive review collected 162 cases [4]. The great majority of these take place in patients who underwent HSCT as treatment for acute leukemias, in some cases for chronic myelogenous leukemia or myelodysplastic syndromes, and for non-malignant diseases in a very few cases. In the review of 41 patients reported by Ruiz-Argüelles and co-workers [3], the latter consisted of three cases of severe aplastic anemia and one of β thalassemia. Of the 64 patients collected in the literature by Wiseman [1] and, in some cases, already included in the review by Ruiz-Argüelles and co-workers [3], there were seven cases of aplastic anemia, one of β thalassemia, one of renal cell carcinoma and one of Langerhans cell histiocytosis for a total of 10 patients without a hematological malignant disorder.

The patient reported here underwent HSCT due to CGD in 2008. The subsequent development of AML in 2010 was unexpected and was immediately recognized via sex-mismatched chromosomes as deriving from donor cells; the patient was male and the donor was his sister. DCL had never been reported in patients with CGD after HSCT. The AML clone showed a translocation, rather common in AML, between chromosomes 9 and 11, with the involvement of the KMT2A (alias MLL) gene (Table 1, Supplementary File S2, Figure S3). The activation of this oncogene is due to fusion in the translocation with the MLLT3 gene, resulting in the production of a fusion protein that leads to the leukemic transformation; this also implies a negative prognosis, particularly in elderly patients [12]. Worthy of note, the sister never developed AML and/or any myeloproliferative disorder during all of the follow-up with the brother, confirming that the t(9;11) in the patient reported here developed in the donor cells after the first HSCT. The presence of the rearrangement was confirmed via FISH of the BM and via RT-PCR of the BM DNA (Table 1). In addition to this anomaly, another translocation was present, namely a balanced translocation between the long arm of chromosome 2 and the long arm of chromosome 7 (Table 1, Supplementary File S2, Figure S3A). This translocation is not recurrent in AML [13]; only one case has been reported with AML and a complex karyotype [14].

Then, the course of the disease was monitored via FISH to establish the proportion of XX and XY cells, thus checking the efficiency of the HSCT, and to detect the presence of the MLL/MLLT3 rearrangement to monitor for the presence of leukemic cells in the BM (Table 1). Analyses using RT-PCR to reveal the rearrangement were always performed in parallel (Table 1).

The results provided evidence of the subsequent relapses and remissions with the two further HSCTs performed in October 2010 and December 2012: again, the donor for the second HSCT was the sister, as in the first HSCT; a non-consanguineous donor was used in the third HSCT. The presence of XX cells with the MLL/MLLT3 rearrangement during relapses suggests that the clone driving the relapse was always the initial one, i.e., the clone of sister’s cells, in which the t(9;11) translocation arose after the first HSCT. This also seems to be true after the third HSCT, even though we do not know the sex of the donor. When the second relapse took place, in fact, in 2012, 98% of the BM cells had XX sex chromosomes, and the molecular rearrangement was present in a molecular analysis (Table 1); after the third HSCT, however, the BM cells were negative for the rearrangement and remained negative until the end of 2014.

The subsequent alarm was in April 2018, with positive molecular results for the MLL/MLLT3 rearrangement. The AML relapsed, and the leukemic clone remained made of XX cells having the MLL rearrangement. Two other chromosome anomalies were now found in addition to the MLL/MLLT3 rearrangement: an unbalanced translocation between the long arm of chromosome 2 and the long arm of chromosome 10, with partial monosomy 2 and partial trisomy 10, and a dicentric isochromosome of the long arm of chromosome 17, idic(17)(p11.2) (Table 1, Supplementary File S2, Figure S3B). No similar translocation has ever been reported in AML, while the isochromosome of the long arm of chromosome 17 is one of the most frequent anomalies in chronic myelocytic leukemia in the blastic or accelerated phase; it has been reported in many patients with acute leukemia, both myeloid and lymphoid, and in several other neoplastic conditions [13]. This isochromosome was already known to be dicentric in most cases [15,16], and in our patient this fact was further demonstrated via a-CGH.

A situation similar to the 2018 relapse then took place after the fourth HSCT with the mother as donor: remission in 2018–2020 and another relapse in February 2020. At this time, the leukemic cells also had XX sex chromosomes with the MLL/MLLT3 rearrangement due to the t(9;11) translocation, but additional anomalies were found: the idic(17)(p11.2) and the unbalanced translocation between chromosomes 2 and 10, both already detected in 2018, as well as a deletion in the short arm of chromosome 12, which had never been found before (Table 1, Supplementary File S2, Figure S3B).

DCL is very rare, and most cases reported have arisen in patients who underwent HSCT due to a neoplastic hematological disease. Very few patients had a non-neoplastic disorder as the primary diagnosis [1,4]. Of the 38 patients reported by Engel and co-workers [17], one had Fanconi anemia and then developed AML in donor cells, and one had sickle cell disease, with chronic myeloid leukemia found as subsequent disease in the donor cells. Our patient had a primary diagnosis of CGD and developed DCL in the form of AML. No other case of CGD has been reported with HSCT and subsequent DCL.

The possible mechanisms in DCL etiology were extensively reviewed by Wiseman [1]. Some of these postulate the presence of either occult leukemia or a predisposing disorder in the donor, and this seems not to be the case of our patient. Other possible mechanisms lie in the effects of previous myeloablative chemotherapy or irradiation of the patient prior to the HSCT, even if this myeloablative-regiment-related possibility is generally considered low [18]. We cannot postulate a mechanism truly suitable for the development of DCL in our patient; the only tenable statement is that DCL is probably a multifactorial process [1].

The results reported here indicate that the AML arose in the donor cells, with the t(9;11) translocation as the primary change, as indicated above. The fact that all subsequent relapses of AML were in XX cells with the MLL/MLLT3 rearrangement strongly suggest that the leukemic clone was always the original one and was able to remain quiescent in the BM during repeated chemotherapy courses, remission periods and HSCT. The negative results in 2013, 2014 and 2018 of the search for the MLL/MLLT3 fusion transcript could be only related to hypo-expression of the mRNA of the fusion gene, and not to the real disappearance of the clone carrying the t(9;11) rearrangement, as the clone reappears in 2015 and 2020. It is important to note that a more reliable technique based on the DNA breakpoints of the fusion genes might be more useful in the detection of the minimal residual disease, as already demonstrated by our group in the case of chronic myeloid leukemia; here, the detection of MRD could only be achieved through patient-specific DNA breakpoint cloning instead of standard RNA-based analysis [19]. Several different additional chromosome anomalies were found since the AML onset (Table 1), and clonal evolution led to clones characterized by various structural anomalies, both infrequent, such as the balanced and unbalanced translocations involving chromosome 2, or recurrent, such as the isochromosome of the long arm of chromosome 17. We can conclude that the leukemic clone arose with the t(9;11) translocation, which was maintained for 10 years, and acquired different additional anomalies, as shown by the results obtained in 2010, 2018 and 2020. It is noteworthy that the XX cells with the MLL/MLLT3 rearrangement were able to remain in the BM for years when cytogenetic and molecular analyses failed to detect them (Table 1). They then repeatedly began to proliferate again, giving rise to the relapses. One remarkable feature could be the presence in the BM of a few normal XY cells after the 2012 HSCT. This was due presumably to the patient’s residual cells or, alternatively, to the third 2012 HSCT donor cells, whose sex is unknown (Table 1). Moreover, a few normal XX cells, even in the BM sample of 2020, could be attributed to the remnants of donor cells, either from the sister or the mother, but are evidently not part of the LMA clone. This kind of cytogenetic course is not very common in AML.

## 5. Conclusions

We can conclude that the leukemic clone arose with the t(9;11) translocation, which was maintained for 10 years, and acquired different additional anomalies, as shown by the results obtained in 2010, 2018 and 2020. It is noteworthy that these XX cells with the MLL/MLLT3 rearrangement were able to remain in the BM for years when cytogenetic and molecular analyses failed to detect them (Table 1). They then repeatedly began to proliferate again, giving rise to the relapses. One remarkable feature could be the presence in the BM of a few normal XY cells, presumably recipient cells, found in analyses performed in 2018 (Table 1), and of a few normal XX cells, even in the BM sample of 2020; these could be the remnants of donor cells, either from the sister or the mother, but are evidently not part of the LMA clone. This kind of cytogenetic course is not very common in AML.

## Figures and Tables

**Table 1:** Results of FISH chromosome analyses to establish the XX/XY chromosome constitution and to detect the presence of the *MLL*/*MLLT3* rearrangement; RT-PCR results for the *MLL*/*MLLT3* rearrangement. Timing of the 1st, 2nd, 3rd, and 4th HSCT is indicated. All analyses are on BM.

Date	BM Karyotype	FISH on BM Nuclei XX/XY	FISH on BM Nuclei *MLL*/*MLLT3*	DNA RT-PCR *MLL*/*MLLT3*
1st HSCT (10/09/2008)				
27/05/2010	46,XX,t(9;11)(p21;q23) [7]/ 46,XX,t(2;7)(q33;q22),t(9;11)(p21;q23) [21]	XX [93%], XY [7%]	positive	positive
21/06/2010	-	XX [75%]	positive [8%]	positive
31/07/2010	46,XX	XX [90%]	negative	positive
02/09/2010	-	XX [92%]	negative	positive
12/10/2010	-	XX [98%]	negative	positive
2nd HSCT (27/10/2010)				
09/02/2011	-	XX [98%]	negative	positive
20/09/2012	-	-	negative	positive
3rd HSCT (10/12/2012)				
19/03/2013	-	-	negative	negative
27/05/2014	-	-	negative	negative
14/12/2014	-	-	-	negative
05/04/2018	-	-	negative	positive
17/04/2018	46,XX,der(2)t(2;10)(q31.3;q23.31),t(9;11)(p21;q23), idic(17)(p11.2) [19]/46,XY [1]	XX [42%], XY [58%] ^a^	positive [42%] ^a^	Positive ^b^
18/05/2018	46,XY [1]	-	negative	-
24/05/2018	46,XY [2]	-	-	-
4th HSCT (27/06/2018)				
31/07/2018	-	-	-	negative
25/09/2018	-	-	-	negative
03/11/2018	-	-	-	negative
12/02/2020	46,XX,der(2)t(2;10)(q31.3;q23.31),t(9;11)(p21;q23), del(12)(p11.2) [4]/46,XX,der(2)t(2;10)(q31.3;q23.31), t(9;11)(p21;q23),del(12)(p11.2),idic(17)(p11.2) [3]/46,XX [4]	-	-	positive

^a^ FISH on mitoses: XX with *MLL*/*MLLT3* [2]; XY with *MLL* not rearranged [17]; the *TP53* gene is deleted in 15 XX mitoses, normally present in XY mitoses. ^b^ The rearrangements/mutations involving genes *CBFβ*/*MYH*, *FLT3* ITD, *NPM1* were also searched for in this BM sample, and the results were negative.

## Data Availability

This study are available on request from the corresponding author. The data are not publicly available due to privacy reason. There are no 3rd party data.

## Supplementary Materials

The following supporting information can be downloaded at: https://www.mdpi.com/article/10.3390/genes14112085/s1. Supplementary File S1: Detailed information on the four HSCT procedures. Supplementary File S2: Figure S1: Production of O_2_^−^ by neutrophils; Figure S2: Western blot analysis of p47^phox^; Figure S3: Chromosome cutoffs; Figure S4: Array comparative genomic hybridization (aCGH), unbalanced chromosome cutoffs.
