# Supplementary material for: Donor Cell Acute Myeloid Leukemia after Hematopoietic Stem Cell Transplantation for Chronic Granulomatous Disease: A Case Report and Literature Review
Source: Genes (Basel). 2023 Nov 16;14(11):2085. doi: 10.3390/genes14112085 (PMC10671685; doi:10.3390/genes14112085)
Supplement: Supplementary file 1 [file genes-14-02085-s001.zip › Supplementary File 1.pdf]

Supplementary file 1: Details on the four HSCT

1 – HSCT 10.09.2008

Conditioning regimen: Fludarabin + Busulfan + Thiotepa

GVHD profilaxis: Cyclosporine

Cells infused (HLA-identical sister as donor): 4.2 x 10^8/kg

Engraftment: 500 neutrophils/µl at day + 15

2 – HSCT 27.10.2010

Conditioning regimen: Aracytine + Melphalan

GVHD profilaxis: Cyclosporine

Cells infused (HLA-identical sister as donor): 3.5 x 10^8/kg

Engraftment: 500 neutrophils/µl on day + 12

3 – HSCT 10.12.2012

Conditioning regimen: Aracytine + Melphalan

GVHD profilaxis: Cyclosporine + ATG

Cells infused (unrelated cord blood donor): 5.2 x 10^7/kg

Engraftment: 500 neutrophils/µl on day + 17

4 – HSCT 27.6.2018

Conditioning regimen: Thiotepa + Threosulfan + Fludarabin + ATG

GVHD prophylaxis: alpha/beta T lymphocyte and CD+19 in vitro depletion

Cells infused (partially matched mother as donor): TCN 2.2x10^8/kg, CD34+ 6.11x10^6/kg, T CD3+ 5.94x10^6/kg, TCR αβ+ 22x10^3/kg, TCR γδ+ 5.94x10^6/kg

Engraftment: day +9, platelet engraftment day +17
