# Supplementary material for: Donor Cell Acute Myeloid Leukemia after Hematopoietic Stem Cell Transplantation for Chronic Granulomatous Disease: A Case Report and Literature Review
Source: Genes (Basel). 2023 Nov 16;14(11):2085. doi: 10.3390/genes14112085 (PMC10671685; doi:10.3390/genes14112085)
Supplement: Supplementary file 1 [file genes-14-02085-s001.zip › Supplementary File 2.pdf]

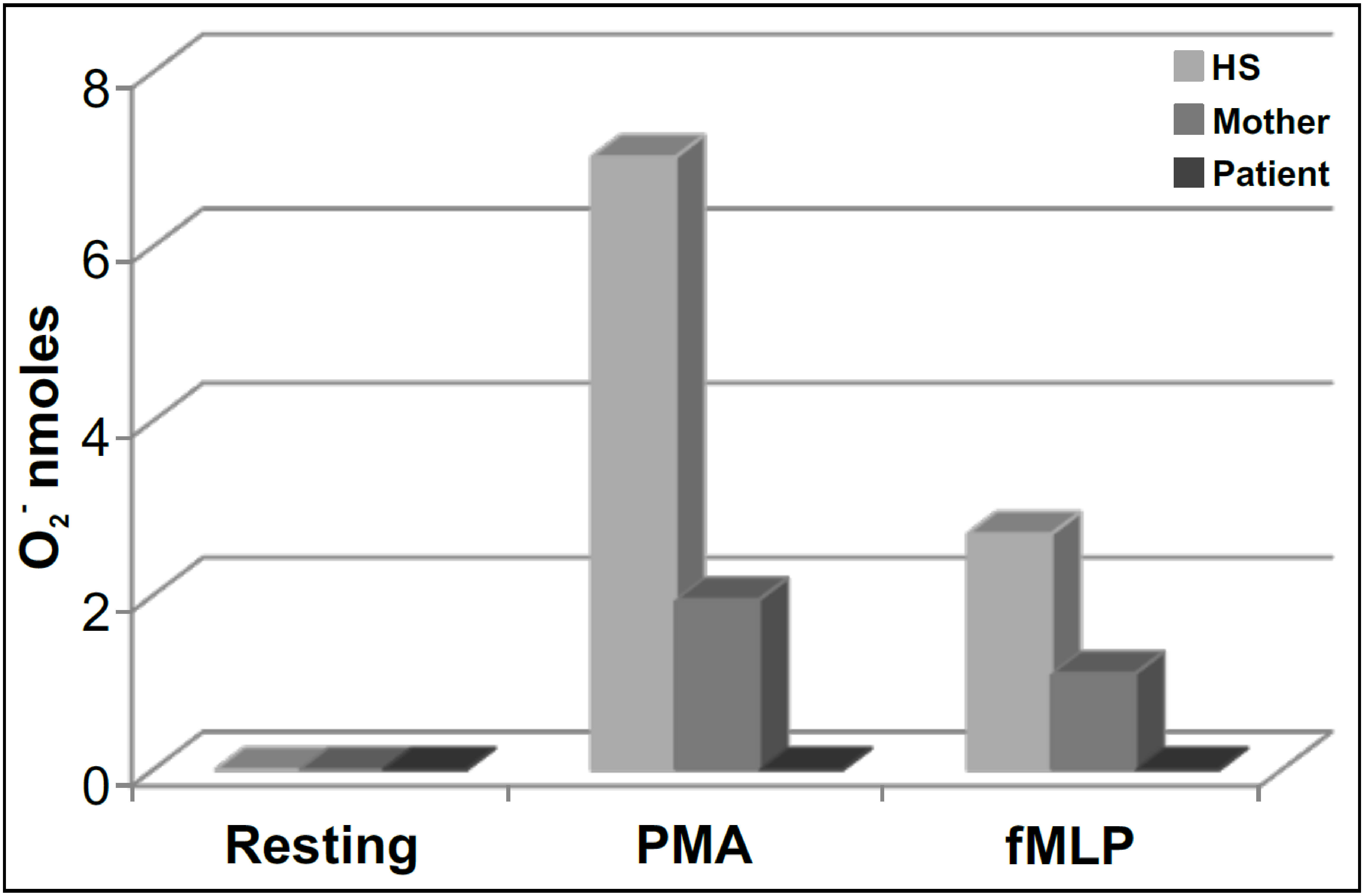

Supplementary Figure S1

Production of O^{2-} by neutrophils. About $2x10^5$ neutrophils of a healthy subject (HS), the patient and his mother suspended in HBSS and stimulated with 100 nM fMLP or 20 ng/mL PMA for 30 minutes. The O^{2-} production was evaluated by cytochrome c reduction.

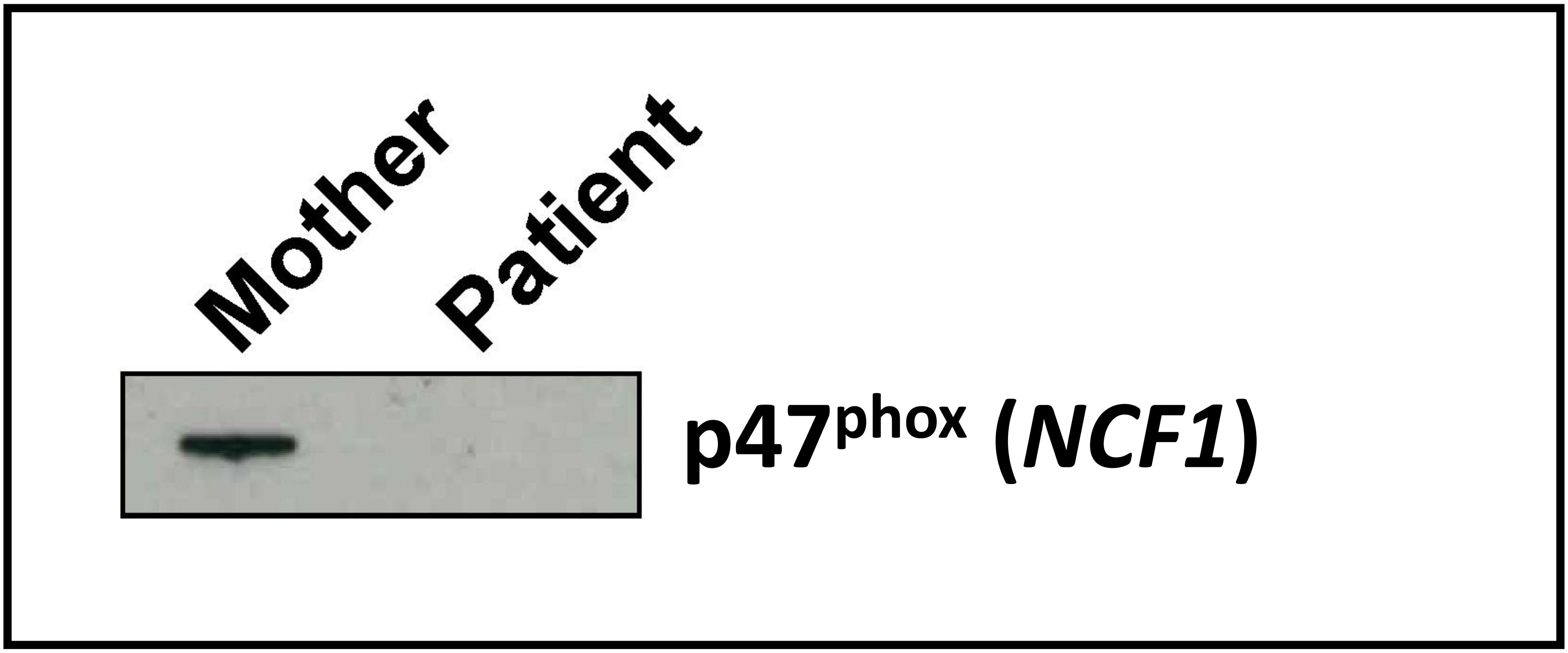

Supplementententary Figure S2
Analysis of p47[phox] expression by Western blot of neutrophils lysates of the patient and his mother with anti-p47[phox] antibody.

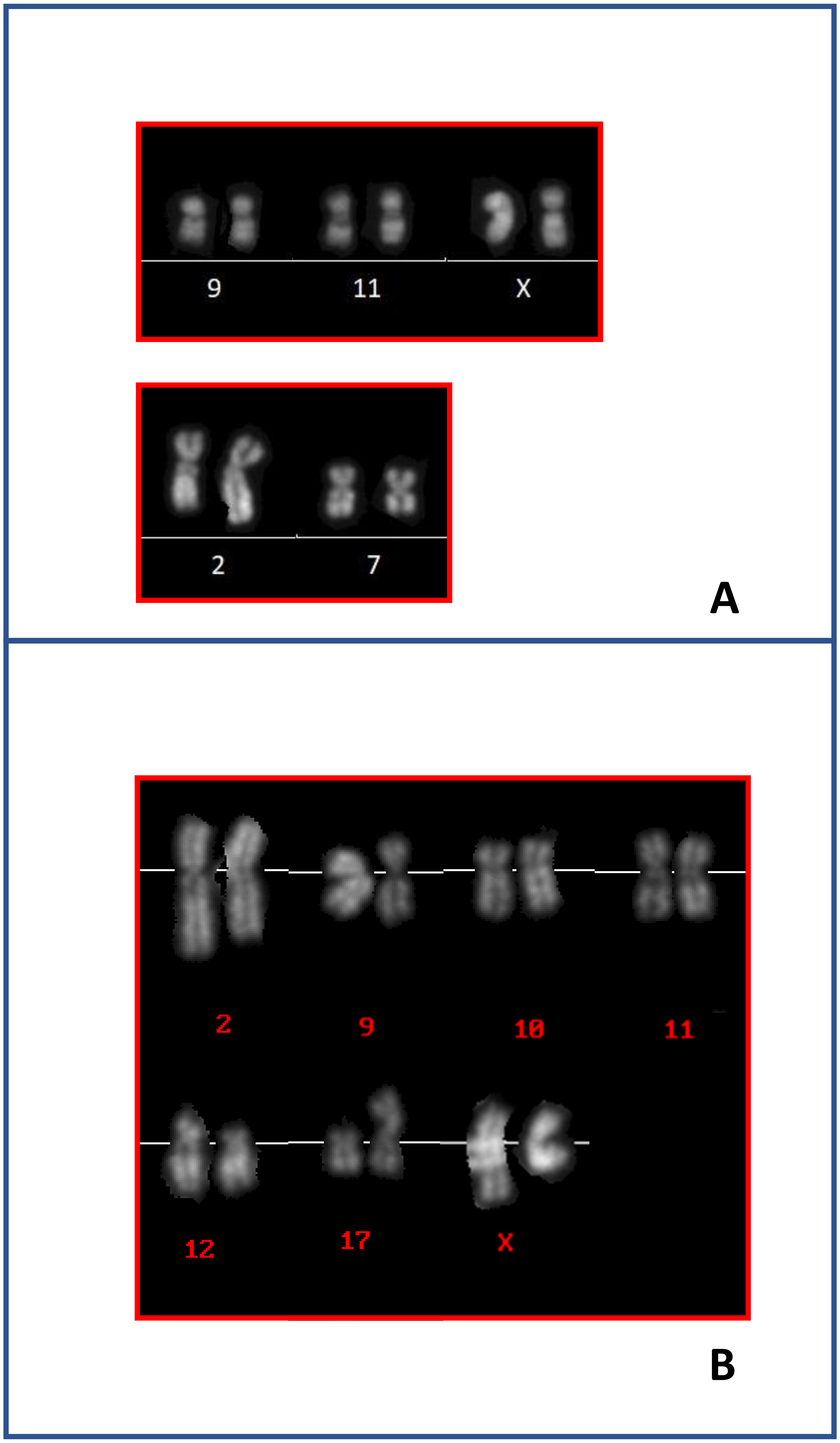

Supplemententary Figure S3

Cut-off of Q-banded chromosomes from a karyotype from the analysis on BM of 2010 (A), showing the translocation t(9;11)(p21;q23), the XX sex chromosomes and, below, the balanced translocation t(2;7)(q33;q22). In B, the cut-off from a karyotype from the analysis of 2018, showing the der(2) chromosome derived by the translocation t(2;10)(q31.3;q23.31), the translocation t(9;11), the normal chromosomes 10, the deletion del(12)(p11.2), the dicentric isochromosome i(17)(p11.2), and the XX chromosomes.

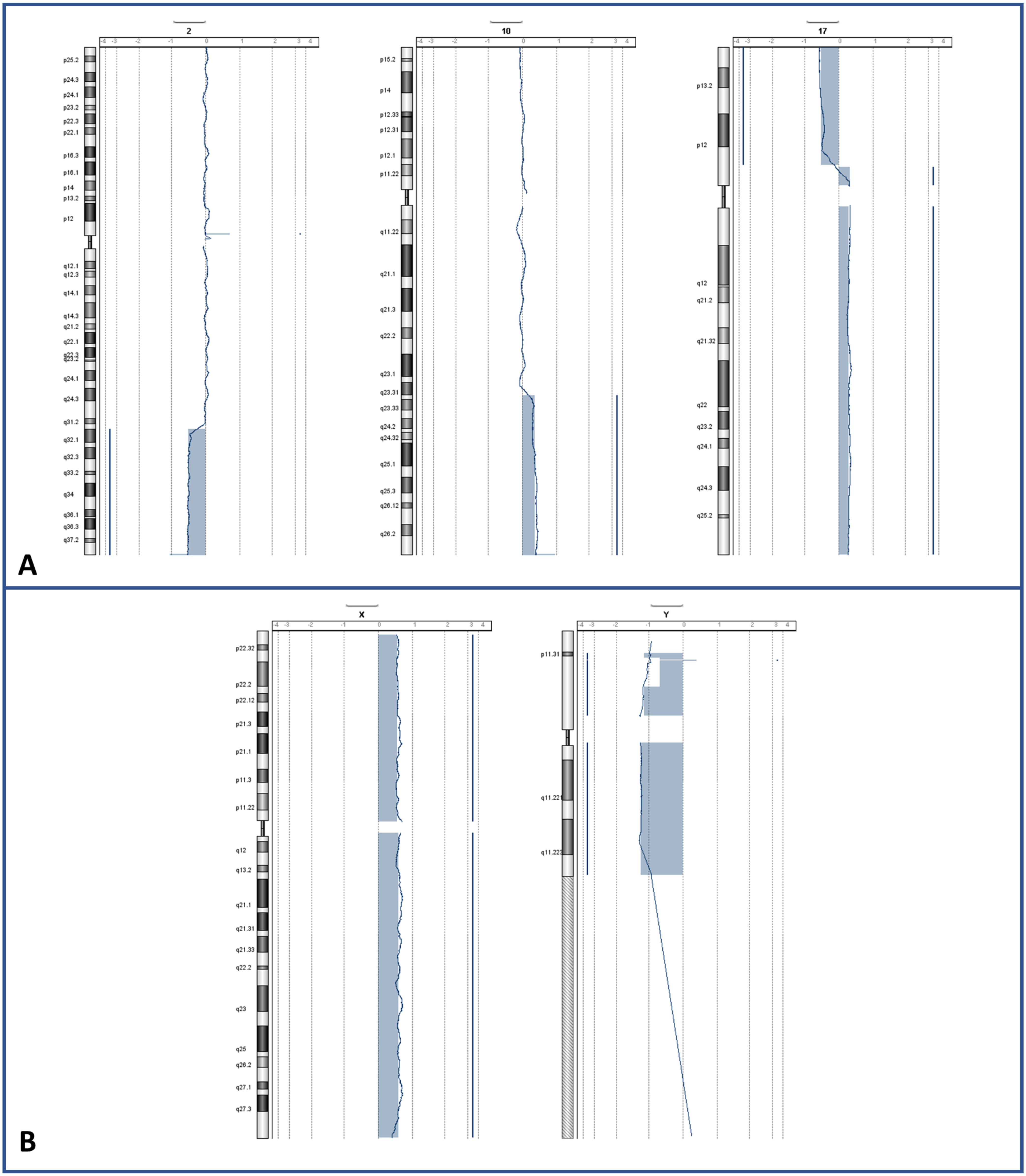

Supplemententary Figure S4

Results of the profiles obtained with aCGH performed in 2018 in DNA from BM. In panel A, the partial monosomy of the long arm of chromosome 2 and the partial trisomy of the long arm of chromosome 10 due to the unbalanced translocation t(2;10)(q31.3;q23.31), and the dicentric isochromosome idic(17)(p11.2). In panel B, the profiles of the chromosomes X and Y, showing that the great majority of the cells have XX sex chromosomes (disomy X in almost all cells and virtually no material of the Y chromosome).
